# 

**Published:** 1889-02-16

**Authors:** 


### CONTENTS.

**Criticism or Cash?** - - - - - - - 307

**Surgery and Therapeutics.**—Curves of the Spine, etc.—Saccharin - - - - - - - 308

**The Doctor's Pulpit.**—"All Sorts and Conditions" of Women - - - - - - - 309

**Literature in Plague Time** - - - - - 310

**The Student's Column** - - - - - - 310

**Health in Our Homes.** By Herman Laurence, M.R.C P., and L.R.C.S.E., etc - - - - - - - 311

**Turning Over New Leaves.**—"Life Without Death" - 312

**Voyages in Vacation.**—XIX. More about Norway - - 313

**Notes and News** - - - - - - - - 314

**Everybody's Page** - - - - - - - - 316

**Annotations.**—"East Wind" Liver—Football Brutalities—Are Abstainers Long-lived? - - - - - - - 317

**Scraps and Gleanings. Amusements and Relaxation** 318

**The Cult of Cleanliness** - - - - - - 319

**False Impressions.** By Caroline Fothergill, Author of "An Enthusiast," "A Voice in the Wilderness," etc. 321

**A Christmas Holiday.**—IV. Brighton Charities - - 322

### EXTRA SUPPLEMENT—THE NURSING MIRROR.

**En Passant.**—Friends in Australia—Nursing at Cairo—A French Nurse—Nurses at Belfast—Provincial News—Jewish Nurses—Another Nurses' Home—An Accident - - lxxvii

**The Pension Fund in the Provinces.**—A Few Errors Corrected - - - - - - - - lxxviii

**Everybody's Opinion.**—A Correction—A Favourite Grievance—A Letter from Jamaica—Wages or Charity?—Nurses as Stewardesses—Catholic Nursing Sisters - - - lxxix

**Examination Questions** - - - - - - lxxx

**Appointments, Vacancies, Notes and Queries** - lxxx

